# Risk Factors for Maxillary Sinus Pathology after Surgery for Midfacial Fracture: A Multivariate Analysis

**DOI:** 10.3390/jcm11216299

**Published:** 2022-10-26

**Authors:** Linli Jiang, Mengsong Wu, Hui Li, Jiayu Liang, Jinlong Chen, Lei Liu

**Affiliations:** 1 State Key Laboratory of Oral Diseases & National Clinical Research Center for Oral Diseases, West China Hospital of Stomatology, Sichuan University, Chengdu 610041, China; 2 Department of Oral and Maxillofacial Surgery, West China Hospital of Stomatology, Sichuan University, Chengdu 610041, China

**Keywords:** maxillofacial injuries, maxillary sinus, internal fracture fixation, multivariate analysis, computed tomography

## Abstract

This study aimed to determine the incidence of maxillary sinus pathology in patients with a midfacial fracture who underwent osteosynthesis surgery and evaluate the associated risk factors. We conducted a retrospective case-control analysis of patients with midfacial fractures involving a maxillary sinus wall who were treated with open reduction and internal fixation (ORIF) between January 2015 and December 2020. Fracture reduction, the number of screws implanted in the maxillary sinus, and the number of screws penetrating the maxillary sinus, etc., were examined as potential risk factors. Maxillary sinus pathology on postoperative CT was considered the primary outcome for case-control analysis. Binary logistic regression was used to identify variables associated with postoperative maxillary sinus pathology. Thereafter, propensity score matching (PSM) was used to extract confounding factors. A total of 262 patients (totaling 372 maxillary sinuses) were included for analysis. PSM yielded 178, 246, and 70 matched sinuses, respectively, depending on the potential risk factors. Postoperative maxillary sinus pathology was visualized in 218 of the 372 maxillary sinuses (58.60%). The risk factors for postoperative maxillary sinus pathology included the number of screws penetrating the maxillary sinus (odds ratio (OR), 1.124; *p* = 0.007), an imperfect maxillary sinus wall fracture reduction (OR, 2.901; *p* = 0.021), and the number of sinus walls involved (OR, 1.383; *p* = 0.011). After PSM, postoperative maxillary sinus pathology was still more prevalent in sinuses with multiple maxillary sinus wall fractures (64.04% vs. 48.31%, *p* = 0.034), sinuses with more screws penetrating the maxillary sinus (64.23% vs. 50.41%, *p* = 0.028), and sinuses with an imperfect reduction (80% vs. 51.43%, *p* = 0.012). In conclusion, maxillary sinus pathology is common after the ORIF of midfacial fractures. Patients with a fracture of multiple maxillary sinus walls require a close follow-up. Screw penetration of the maxillary sinus should be avoided to prevent maxillary sinus pathology after a midfacial fracture ORIF.

## 1. Introduction

Midfacial fractures account for 45.4% to 79.1% of all facial fractures and can seriously affect patient appearance and function [1,2,3,4,5]. The midfacial region is defined based on the resulting AO CMF classification system within anatomical regions: zygoma, upper central midface, intermediate central midface, lower central midface, frontal, parietal, sphenoidal, and temporal [6]. The involvement of the maxillary sinus walls is frequent. Sinusitis is the most common disorder of the maxillary sinus after trauma [7,8,9,10]. The symptoms of maxillary sinusitis include nasal congestion, nasal discharge, facial pain, rhinocnesmus, etc., [11,12]. Acute maxillary sinusitis can lead to life-threatening complications while chronic disease can seriously affect quality of life because of recurrent episodes and related discomfort [13,14,15]. Decreasing the incidence of maxillary sinusitis has become a goal of facial fracture management.

Many researchers have focused on the pathogenesis of post-traumatic maxillary sinusitis to explore possible risk factors. Cengiz et al. suggested that hematocele and maxillary sinus fractures can cause sinus outflow obstruction and cause long-term sinusitis [16]. Ballon et al. indicated that tissue or fracture fragment herniation into the sinus may be related to post-traumatic sinusitis-associated discomfort [17]. This complication can also be provoked iatrogenically. The separation of bones around the maxillary sinus, changes in the clearance mechanism of the maxillary sinus, and hematocele formation in the sinus cavity when performing Le Fort I osteotomy can cause inflammatory changes in the maxillary sinus [18,19,20], which can also occur after the reduction of midfacial fractures. In addition, Elhamruni showed that dental implants that penetrate the maxillary sinus by >3 mm can cause inflammatory changes in the sinus [21]. Similarly, the screws used in open reduction and internal fixation (ORIF) procedures for midfacial fracture repair often penetrate the maxillary sinus. However, no studies have been performed to investigate the risk factors for maxillary sinusitis after the ORIF of midfacial fractures.

Maxillary sinus pathology can be evaluated objectively by assessing characteristics such as mucosal thickening, gas–fluid level, and maxillary sinus opacity on computed tomography (CT) [22,23,24]. This study aimed to determine the incidence of maxillary sinus pathology in the CT of patients with a midfacial fracture involving a maxillary sinus wall who underwent ORIF surgery and to evaluate the associated risk factors.

## 2. Materials and Methods

### 2.1. Ethical Considerations

The study followed the rules of the Declaration of Helsinki and was approved by the Institutional Review Board of the West China Hospital of Stomatology, Sichuan University (protocol code WCHSIRB-D-2021-299).

### 2.2. Study Population

This hospital-based retrospective case-control study examined the medical and radiological records of patients diagnosed with a midfacial fracture involving a maxillary sinus wall who underwent ORIF surgery using titanium plates and screws in our department between January 2015 and December 2020. Based on the Kendall rule and events per variable (EPV) theory [25,26], the theoretical sample size is as least 90. All patients underwent CT before surgery and had at least 3 months of postoperative CT follow-up. We excluded patients with a history of preoperative sinusitis and/or rhinitis and those with obvious dental lesions (e.g., periapical disease, advanced periodontal disease, and oroantral communication following dentoalveolar surgery) or pre-existing maxillary sinus pathology on CT.

### 2.3. Factors of Interest and Data Collection

Factors of interest included age, gender, history of diabetes, number of sinus walls involved, preoperative CT evaluation of maxillary sinus, the presence or absence of endotracheal intubation, number of screws implanted in the maxillary sinus wall, number of screws penetrating the maxillary sinus, and fracture reduction. Age, gender, history of diabetes, and the presence or absence of endotracheal intubation were collected from medical records. The number of sinus walls involved and preoperative CT evaluations of the maxillary sinus were evaluated by preoperative CT. The number of screws implanted in the maxillary sinus wall, the number of screws penetrating the maxillary sinus, and fracture reduction were evaluated by postoperative CT.

The patients were classified as children (under 18), adults (18–59 years), and the aged (60 and older than 60).

The number of sinus walls involved ranged from 1 to 5 (including the anterior wall, posterior wall, superior wall, medial wall, and floor).

**Figure 1:**
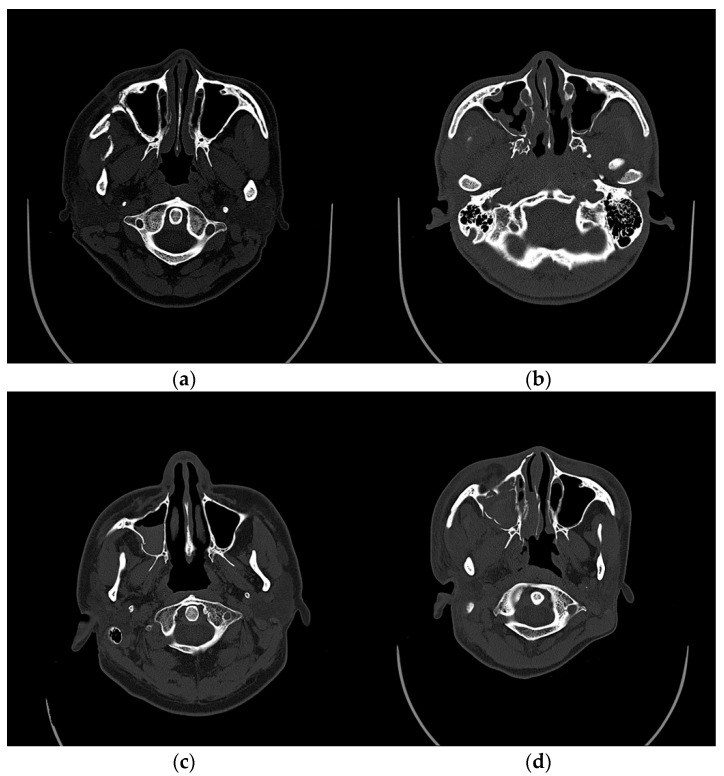
Representative CT sections showing the different preoperative evaluations of the maxillary sinus. (**a**) normal; (**b**) maxillary mucosal thickening; (**c**) gas–fluid level; (**d**) total opacification.

Preoperative CT evaluations of the maxillary sinus were classified as normal, mucosal thickening, gas–fluid level, and total opacification based on Rouby’s classification and Gliklich Metson’s classification (Figure 1) [22,23,24]. As for the mucosa thickening, the author used the criterion of Sanchez-Perez et al., in which the thickening of the mucosa from 2 mm was considered pathological [27]. This was considered only when the mucosa thickening location was not related to obvious dental lesions. The presence of gas–fluid in the sinus was evaluated based on the presence of a fluid opacification, noted on the CT axial scan image, according to the criterion of Bomeli [28]. The presence of total opacification in the sinus was noted on the CT axial, sagittal, and coronal scan images.

**Figure 2:**
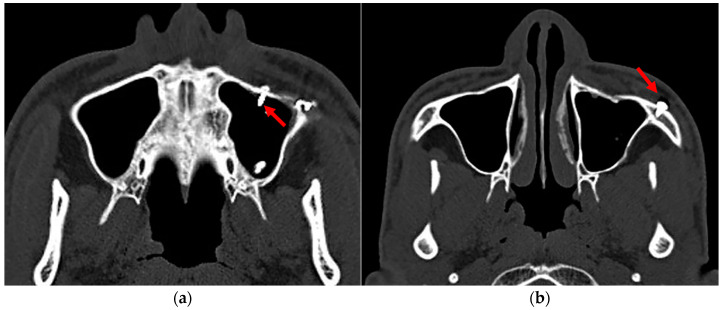
Implant penetration into the maxillary sinus. (**a**) The arrow indicates the implants (screws) penetrated the maxillary sinus. (**b**) The arrow indicates the implants (screws) within the maxillary sinus wall.

Considering the medial and posterior walls of the maxillary sinus were unapproachable and inaccessible during ORIF, the screws implanted in the anterior wall, superior wall, and floor were counted as the number of screws implanted in the maxillary sinus wall. And once these screws penetrated through the bone wall of the maxillary sinus, they were counted as the number of screws penetrating the maxillary sinus (Figure 2).

**Figure 3:**
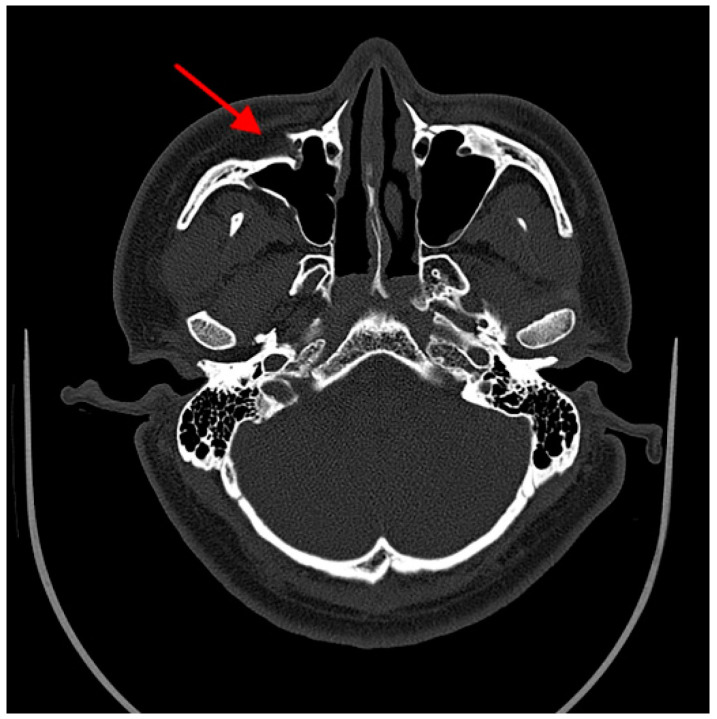
Imperfect reduction. Axial scan through the superior portion of the maxillary sinus and zygomatic arch. The arrow indicates the imperfect reduction of the maxillary sinus wall.

Maxillary sinus wall fracture reduction evaluation was based on Ellis’s classification [29]. Any asymmetry on the images more than 2 mm in magnitude was considered an imperfect reduction of the fracture on the CT axial scan image (Figure 3).

Maxillary sinus pathology including mucosal thickening, gas–fluid level, and maxillary sinus opacity on the postoperative CT was considered the primary outcome for the case-control analysis.

All patients’ preoperative and postoperative CT data were acquired from the CT scanner (Philips, Best, The Netherlands). The obtained CT images were viewed using the PACS (Picture Archiving and Communication System) giving a three-dimensional reconstruction module and the multiplanar reformations module, i.e., axial, sagittal, and coronal slices. All images were assessed under standardized conditions at the same examination place. Radiographic examinations were assessed by two investigators, who followed our standardized clinic protocol, and their assessments were tested for consistency.

### 2.4. Statistical Analysis

Statistical analyses were performed using SPSS software version 22.0 (IBM Corp., Armonk, NY, USA). The presence and/or extent of each factor of interest was assigned a value as shown in Table 1. The analysis unit in the study was the sinus. Descriptive data are presented as means with standard deviations. Binary logistic regression was used to identify variables associated with post-traumatic maxillary sinus pathology after adjusting for confounding variables. A logistic regression was implemented and the criteria used for the entry and removal variables were 0.05 and 0.1, respectively, in the final model. Considering that in a logistic model, even if a factor is not found to be significantly different, if it is a confounding factor, the propensity score matching (PSM) will increase the statistical precision and improve the quality of the study. In order to further remove the potential bias of confounding factors, a PSM was performed to verify the above results. A 1:1 PSM method was used, within propensity score calipers of ±0.002 SD [30]. Further, a post-matching logistic regression analysis was conducted to check whether the distribution of potential confounding variables was equal in both groups. Finally, the chi-square test was performed to verify the correlation between post-traumatic maxillary sinus pathology and three potential risk factors, respectively, (number of sinus walls involved, number of screws penetrating the maxillary sinus, and the reduction of the sinus wall); *p*-values lower than 0.05 were considered statistically significant. Results are presented as odds ratios (ORs) with 95% confidence intervals (CIs). The kappa statistic was used to evaluate interrater reliability; *p* <0.05 was considered significant.

## 3. Results

A total of 285 patients were eligible for study inclusion. Twenty-three were excluded based on the criteria. Therefore, 262 patients (totaling 372 maxillary sinuses) were included for analysis. Fractures were bilateral in 152 patients and unilateral in 110. One hundred ninety-eight patients were men (75.57%) and 64 were women (24.43%). The mean age was 35.79 ± 14.13 years (range, 3–67).

Based on postoperative CTs, 218 of 372 maxillary sinuses (58.60%) exhibited post-traumatic maxillary sinus pathology. Among these, mucosal thickening was most common (52.15%), followed by maxillary sinus opacity (5.37%), and gas–fluid level (1.08%). Patient and radiological characteristics are shown in Table 2.

The results of the binary logistic regression evaluation of risk factors for post-traumatic maxillary sinus pathology were shown in Table 3. Three factors were associated with increased odds of post-traumatic maxillary sinus pathology: the number of screws penetrating the maxillary sinus (OR, 1.124; *p* = 0.007), an imperfect maxillary sinus wall fracture reduction (OR, 2.901; *p* = 0.021), and the number of sinus walls involved (OR, 1.383; *p* = 0.011). Other potential risk factors were not statistically significant.

The above results showed that three risk factors were statistically significant (the number of sinus walls involved, the number of screws penetrating the maxillary sinus, and the fracture reduction of the maxillary sinus wall). Based on this result, a PSM was performed to further remove the potential bias of confounding factors. The above three risk factors with statistical significance were set as the group indicators of the PSM, and all other factors were set as confounding factors. First of all, the 1:1 PSM procedure yielded 178, 246, and 70 matched sinuses, respectively, depending on the above-mentioned three risk factors. Then, three post-matching logistic regression analyses were performed to check whether the distribution of the potential confounding variables was equal in the different groups. As shown in Table 4, all confounding factors were balanced in different groups (the number of sinus walls involved (≤3) vs. the number of sinus walls involved (>3), the number of screws penetrating the maxillary sinus (≤4) vs. the number of screws penetrating the maxillary sinus (>4), and imperfect reduction vs. perfect reduction). Finally, three chi-square tests were performed, respectively, to verify whether these three risk factors were statistically significant after adjusting for confounding factors (Table 5). The results showed that after the PSM, postoperative maxillary sinus pathology was still more prevalent in sinuses with multiple maxillary sinus walls fractured (64.04% vs. 48.31%, *p* = 0.034), sinuses with more screws penetrating the maxillary sinus (64.23% vs. 50.41%, *p* = 0.028), and sinuses with an imperfect reduction (80% vs. 51.43%, *p* = 0.012).

The kappa statistic for the two radiography examiners was 0.813 (*p* = 0.000), indicating high interobserver agreement.

## 4. Discussion

Post-traumatic maxillary sinusitis is a common complication of midfacial fractures that can be detected by CT. Although surgeons are concerned about maxillary sinus pathology after these fractures, few clinical studies have investigated the associated risk factors. To our knowledge, this is the first large-scale study to examine the incidence of maxillary sinus pathology in patients with surgically repaired midfacial fractures and perform a multivariate analysis to determine the associated risk factors.

Previous studies have focused on the prevalence of maxillary sinus pathology in the non-traumatic population and reported rates ranging from 37.2% to 45.1% [31,32]. Aksoy, Manor, and Penarrocha-Oltra et al. found that a history of sinusitis and dental lesions were associated with maxillary sinus pathology [33,34,35]. Considering that the main purpose of this study was to investigate traumatic and iatrogenic factors contributing to maxillary sinus pathology, we excluded patients with a history of sinusitis or obvious dental lesions. Even so, maxillary sinus pathology was present in 218 of 372 maxillary sinuses imaged (58.60%), showing that maxillary sinus pathology is common after the ORIF of midfacial fractures. This suggests that clinical attention should be paid to the condition of the maxillary sinus in these patients.

Considering such a high prevalence of maxillary sinus pathology after surgery for midfacial fracture, the risk factors for this symptom are worth discussing. Although no systematic study has been performed to discuss risk factors for post-traumatic maxillary sinus pathology, there have been some similar studies in the non-traumatic field. Dentistry studies have determined that dental implants penetrating the maxillary sinus are associated with maxillary sinus pathology [20,36]. Given that the screws used in ORIF procedures often penetrate the maxillary sinus, we examined the number of titanium screws penetrating the maxillary sinus as a potential risk factor. Not surprisingly, this factor was shown to increase the risk of postoperative maxillary sinus pathology either in the entire study population or in a propensity score-matched population. Despite the well-known acknowledgment that surgeons tend to avoid penetrating screws into the maxillary sinus when performing ORIF of midfacial fractures, no evidence exists, and thus this study further verified this viewpoint for the first time. We suggest that screws should be placed in regions of thicker bone, represented by the horizontal and vertical buttresses. Furthermore, bone thickness at the horizontal and vertical buttresses should be measured before surgery to select screws of the appropriate length to avoid maxillary sinus penetration [37].

The imperfect reduction of the maxillary sinus wall also increased the risk of postoperative maxillary sinus pathology in our study. Previous studies have illustrated that the most important goals of midfacial fracture treatment include the correction of malocclusion, improvement of poor facial aesthetics, restoration of midfacial height, and repair of midface projection [38]. The accurate reduction of the maxillary sinus wall has not been an established treatment goal and is often ignored. However, our results emphasize the importance of accurate reduction, as an imperfect reduction was associated with maxillary sinus pathology. Therefore, surgeons should attempt to accurately reduce maxillary sinus wall fractures when performing ORIF procedures.

In the present study, we also found that a greater number of sinus walls involved in the fracture increased the risk of post-traumatic maxillary sinus pathology, as would be expected. A greater number of involved sinus walls indicates a more severe injury and a higher possibility of fracture-associated mucosal laceration in the sinus and displacement of fracture fragments. This finding implies that patients with a fracture of multiple maxillary sinus walls require close follow-up.

Considering that this was a retrospective study that was not randomized, we combined binary regression analysis with PSM to provide a control group very similar to what may be achieved in a randomized controlled trial. PSM methods provide certain advantages over more traditional logistic regression methods to control confounding factors to generate an unbiased control group enabling the exploration of causal relationships using observational data [39]. According to this method, the results after PSM further verified the results of the logistic regression before PSM.

There remain some limitations in this study, such as a retrospective and single-center study, etc. As a result, selection bias may still exist. Although it has been confirmed that the prevalence of maxillary sinus pathology was high in patients after ORIF of midfacial fractures and the risk factors for that included the number of titanium screws penetrating the maxillary sinus, an imperfect reduction, and the number of sinus walls involved. The results indicated that a random controlled trial or a multicenter and large sample study is necessary to further verify this conclusion.

## 5. Conclusions

In conclusion, maxillary sinus pathology is common after the ORIF of midfacial fractures. This suggests that clinical attention should be paid to the condition of the maxillary sinus in these patients, especially patients with a fracture of multiple maxillary sinus walls. When performing ORIF for midfacial fractures, screw penetration of the maxillary sinus should be avoided and sinus wall fractures should be reduced as accurately as possible to prevent post-traumatic maxillary sinus pathology.

## Figures and Tables

**Table 1:** Variables and assignment of values.

Variables	Value
Maxillary sinus pathology (Y):	
No	0
Yes	1
Age group (X1):	
0–17	0
18–59	1
>60	2
Gender (X2):	
Male	0
Female	1
History of diabetes (X3):	
No	0
Yes	1
Number of sinus walls involved (X4):	
Preoperative CT evaluation of maxillary sinus (X5):	
Normal	0
Mucosal thickening	1
Air–fluid level	2
Total opacification	3
The presence or absence of endotracheal intubation (X6)	
Endotracheal intubation	0
Tracheostomy tube	1
Number of screws implanted in the maxillary sinus (X7)	
Number of screws penetrating the maxillary sinus (X8)	
Fracture reduction of maxillary sinus wall (X9)	
Perfect	0
Imperfect	1

CT, computed tomography.

**Table 2:** Patient and radiological characteristics.

Variables	Post-Traumatic Maxillary Sinus Pathology	
	No	Yes
Age (patients):		
0–17	5	17
18–59	104	123
>60	4	9
Gender (patients):		
Male	83	115
Female	30	34
History of diabetes (patients):		
No	108	145
Yes	5	4
Number of sinus walls involved (sinus)	3.30 ± 1.07	3.76 ± 0.98
Preoperative CT evaluation of maxillary sinus (sinus):		
Normal	13	6
Mucosal thickening	61	89
Air–fluid level	24	25
Total opacification	56	98
The presence or absence of endotracheal intubation (sinus):		
Endotracheal intubation	148	204
Tracheostomy tube	6	14
Number of screws implanted in the maxillary sinus (sinus)	11.94 ± 5.37	13.30 ± 5.38
Number of screws penetrating the maxillary sinus (sinus)	4.52 ± 2.83	5.56 ± 3.07
Fracture reduction of maxillary sinus wall (sinus):		
Perfect	147	188
Imperfect	7	30

CT, computed tomography.

**Table 3:** Binary logistic regression evaluation of risk factors for post-traumatic maxillary sinus pathology.

Factors	Variables	*p*-Value	OR	95% CI
Age	X1	0.062		
18–59		0.023	0.325	0.123 to 0.856
>60		0.295	0.475	0.118 to 1.915
Gender (1)	X2	0.786	0.928	0.541 to 1.591
History of diabetes	X3	0.390	0.613	0.201 to 1.869
Number of sinus walls involved	X4	**0.011**	1.383	1.077 to 1.776
Preoperative CT evaluation of maxillary sinus	X5	0.486		
Mucosal thickening (1)		0.187	2.132	0.693 to 6.558
Air–fluid level (2)		0.538	1.463	0.436 to 4.909
Total opacification (3)		0.301	1.854	0.575 to 5.978
The presence or absence of endotracheal intubation	X6	0.320	1.707	0.595 to 4.894
Number of screws implanted in the maxillary sinus	X7	0.966	0.999	0.952 to 1.048
Number of screws penetrating the maxillary sinus	X8	**0.007**	1.124	1.033 to 1.222
Fracture reduction of maxillary sinus wall (1)	X9	**0.021**	2.901	1.176 to 7.160

OR, odds ratio; CI, confidence interval; CT, computed tomography; *p*-values lower than 0.05 were considered statistically significant. Significant *p*-values were bold.

**Table 4:** Patients’ potential confounding variables in different groups based on potential risk factors.

Number of Sinus Walls Involved			Number of Screws Penetrating the Maxillary Sinus			Fracture Reduction of Maxillary Sinus Wall		
**Variables**	**OR**	***p*-Value**	**Variables**	**OR**	***p*-Value**	**Variables**	**OR**	***p*-Value**
Age	-	0.907	Age	-	0.936	Age	-	0.592
Gender	1.207	0.604	Gender	1.099	0.755	Gender	0.575	0.483
History of diabetes	0.930	0.946	History of diabetes	1.077	0.959	History of diabetes	1.276	0.875
Preoperative CT evaluation of maxillary sinus	-	0.964	Number of sinus walls involved	0.957	0.887	Number of sinus walls involved	0.908	0.891
The presence or absence of endotracheal intubation	1.311	0.661	Preoperative CT evaluation of maxillary sinus	-	0.999	Preoperative CT evaluation of maxillary sinus	-	0.932
Number of screws implanted in the maxillary sinus	1.131	0.701	The presence or absence of endotracheal intubation	1.470	0.632	The presence or absence of endotracheal intubation	2.062	0.617
Number of screws penetrating the maxillary sinus	0.995	0.990	Number of screws implanted in the maxillary sinus	1.136	0.694	Number of screws implanted in the maxillary sinus	0.727	0.589
Fracture reduction of the maxillary sinus wall	0.471	0.322	Fracture reduction of the maxillary sinus wall	0.622	0.332	Number of screws penetrating the maxillary sinus	0.474	0.187

OR, odds ratio; CT, computed tomography; *p*-values lower than 0.05 were considered statistically significant.

**Table 5:** Evaluation of risk factors for post-traumatic maxillary sinus pathology before and after PSM.

Factors	Pre-Matching		Propensity Score Matching	
	OR (95%CI)	*p*-Value	Chi-Square Value	*p*-Value
Number of sinus walls involved	1.383 (1.077 to 1.776)	**0.011**	4.473 (64.04% vs. 48.31)	**0.034**
Number of screws penetrating the maxillary sinus	1.124 (1.033 to 1.222)	**0.007**	4.802 (64.23% vs. 50.41%)	**0.028**
Fracture reduction of maxillary sinus wall (1)	2.901 (1.176 to 7.160)	**0.021**	6.341 (80% vs. 51.43%)	**0.012**

OR, odds ratio; CI, confidence interval. *p*-values lower than 0.05 were considered statistically significant. Significant *p*-values were bold.

## Data Availability

Not applicable.
